# Simultaneous Quantification of Three Curcuminoids and Three Volatile Components of *Curcuma longa* Using Pressurized Liquid Extraction and High-Performance Liquid Chromatography

**DOI:** 10.3390/molecules23071568

**Published:** 2018-06-28

**Authors:** In-Cheng Chao, Chun-Ming Wang, Shao-Ping Li, Li-Gen Lin, Wen-Cai Ye, Qing-Wen Zhang

**Affiliations:** 1 State Key Laboratory of Quality Research in Chinese Medicine, Institute of Chinese Medical Sciences, University of Macau, Macao 999078, China; yqzhou1986@gmail.com (I.-C.C.); cmwang@umac.mo (C.-M.W.); spli@umac.mo (S.-P.L.); ligenl@umac.mo (L.-G.L.); 2 Institute of Traditional Chinese Medicine and Natural Products, Jinan University, Guangzhou 510632, China; chywc@aliyun.com

**Keywords:** HPLC, *Curcuma longa*, turmeric, curcuminoids, turmerone, quantification

## Abstract

A high-performance liquid chromatography (HPLC) method was investigated for the simultaneous quantification of two chemical types of bioactive compounds in the rhizome of *Curcuma longa* Linn. (turmeric), including three curcuminoids: Curcumin, bisdemethoxycurcumin, and demethoxycurcumin; and three volatile components: *ar*-turmerone, *β*-turmerone, and *α*-turmerone. In the present study, the sample extraction system was optimized by a pressurized liquid extraction (PLE) process for further HPLC analysis. The established HPLC analysis conditions were achieved using a Zorbax SB-C18 column (250 mm × 4.6 mm i.d., 5 μm) and a gradient mobile phase comprised of acetonitrile and 0.4% (*v*/*v*) aqueous acetic acid with an eluting rate of 1.0 mL/min. The curcuminoids and volatile components were detected at 430 nm and 240 nm, respectively. Moreover, the method was validated in terms of linearity, sensitivity, precision, stability and accuracy. The validated method was successfully applied to evaluate the quality of twelve commercial turmeric samples.

## 1. Introduction

*Curcuma longa* Linn. (*C. longa*), belonging to the Zingiberaceae family, is a native plant of southern Asia, mainly India and China. The rhizome of *C. longa*, also called turmeric, has long been used as a spice or food additive and in traditional medicine system in Asia. In traditional Chinese medicine, it is called “Jianghuang”, and has been widely used for the treatment of diabetic wounds, hepatic disorders and cardiovascular disease [1]. Phytochemical investigation of turmeric has revealed it contains curcuminoids and volatile oils as the major components [2]. Curcumin and two demethoxy derivatives, demethoxycurcumin and bisdemethoxycurcumin, are the major curcuminoids in turmeric, which have anti-cancer, anti-inflammatory, neuroprotective, anti-Alzheimers and anti-oxidant activities [2,3,4,5,6,7,8]. Curcuminoids have always been the focus of drug research. Furthermore, the volatile oil of turmeric is also widely used in cosmetic and health products, and possesses antimicrobial, antifungal, and antiarthritic activities [9,10,11]. Recently, studies have indicated that turmerones were the active constituents in turmeric oil, and proved their anti-cancer, anti-inflammatory, antiplatelet, anti-angiogenic, and neuropharmacological properties [12,13,14,15,16]. Therefore, both curcuminoids and volatile components accounted for the efficacy of turmeric. As such, both kinds of components should be used as markers for an evaluation of the quality of turmeric and the products from turmeric. Many methods, including HPLC or ultra performance liquid chromatography (UPLC) coupled with UV-vis and/or MS detector [17,18,19,20], high performance thin layer chromatography (HPTLC) [21], capillary electrophoresis (CE) [22], and microemulsion electrokinetic chromatography [23], have been developed for qualitative and quantitative analysis of curcuminoids in turmeric and its pharmaceutical preparations. Gas chromatography-flame ionization detector (GC-FID) and gas chromatography–mass spectrometry (GC-MS) are the conventional methods used for the analysis of the volatile constituents of turmeric. *ar*-Turmerone, *α*-turmerone, and *β*-turmerone were determined to be the major compounds of the volatile oil [24,25]. However, the content of volatile components is only determined by the percentage of the selected ions peak area. Up till now, no method was reported for simultaneous quantitative analysis of major curcuminoids and volatile components in turmeric.

**Figure 1:**
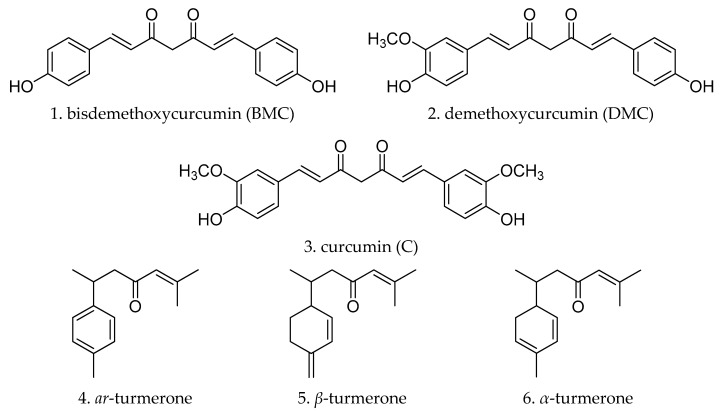
Structures of three curcuminoids and three volatile compounds in turmeric.

In this paper, an HPLC method coupled with pressurized liquid extraction (PLE) was developed for simultaneous quantification of the two classes of bioactivity components in turmeric, including three curcuminods: Bisdemethoxycurcumin, demethoxycurcumin and curcumin; and three volatile components: *ar*-turmerone, *β*-turmerone and *α*-turmerone (Figure 1). The developed method was successfully applied to evaluate the quality of twelve samples of turmeric.

## 2. Results and Discussion

### 2.1. Optimization of PLE Procedure

PLE has become a green sample preparation method for plant analysis due to its advantages of good repeatability, shorter extraction time, lower extraction solvent assumption, and higher extraction efficiency [26,27,28]. Schieffer [29] found that the contents of curcuminoids from PLE were higher than those from Soxhlet extraction and single or multiple ultrasonic extractions. PLE was also used for the extraction of volatile compounds in several Curcuma plants [25,30]. Thus, PLE was adopted for the extraction of both curcuminoids and volatile components in turmeric in the present research.

The PLE procedure was optimized using a univariate approach. All variables involved in the procedure have been assayed: Solvent (methanol, ethanol, 50% methanol, 50% ethanol), temperature (80–160 °C), particle size (0.125–0.45 mm), static extraction time (5, 10, 15 min), and extraction cycles (1, 2, 3 cycles). Total peak areas of the six investigated compounds were used as a marker for evaluation of the extraction efficiency. According to the results of the optimization, the final conditions of the PLE method were: solvent, ethanol; temperature, 100 °C; particle size, 0.20–0.30 mm; static extraction time, 5 min; static cycle, 1; pressure, 1500 psi, and 60% of the flush volume.

### 2.2. Optimization of Chromatographic Conditions

**Figure 2:**
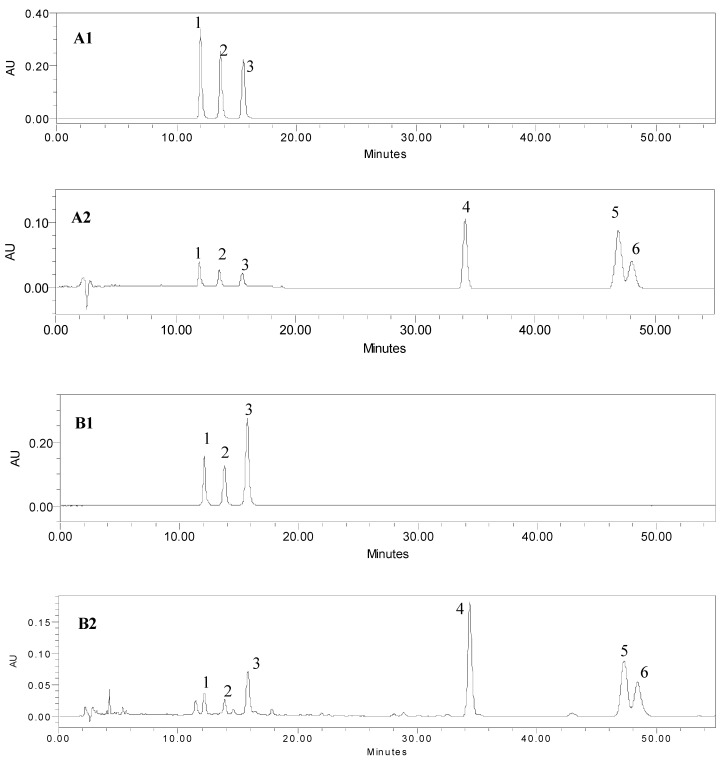
HPLC chromatograms of the mixed standards solution (**A1**: 430 nm, **A2**: 240 nm) and turmeric sample (**B1**: 430 nm, **B2**: 240 nm). The peaks were numbered the same as those in Figure 1.

The optimization of the HPLC conditions was performed using the mix reference compound solution and sample 8. Many HPLC methods have been developed for analyzing the three curcuminoids [17,18], and the LC-MS method has been reported to identify the characteristic curcuminoids and sesquiterpenoids in turmeric [31]. However, we found that *β*-turmerone and *α*-turmerone were hard to separate. Therefore, different types of columns, including C18 (Zorbax SB-C18 and Zorbax Extend-C18), Phenyl (Zorbax SB-Phenyl), and C8 (Phenomenex Luna C8), were tested. It was found that *β*-turmerone and *α*-turmerone could be separated only on a Zorbax SB-C18 column among those tested columns. Different compositions of the mobile phase (methanol-water, acetonitrile-water, and acetonitrile-acid aqueous solution) and different column temperatures were also tested. As a result, acetonitrile and 0.4% aqueous acetic acid was chosen as the eluting solvent to achieve a better resolution and an acceptable tailing factor. It was also found that an increasing column temperature could improve the chromatographic behavior. The resolution increased and the retention time shortened with an increasing column temperature. At the column temperature of 35 °C, all six compounds were well separated (Figure 2). For the selection of the detection wavelengths, wavelengths from 200 to 800 nm were scanned. The UV maximum absorption wavelengths were chosen to monitor these analytes, i.e., 430 nm for the three curcuminoids and 240 nm for the three volatile compounds.

### 2.3. HPLC Method Validation

#### 2.3.1. Calibration, Limits of Detection, and Quantification

The calculated results are given in Table 1. The calibration curves of the six analytes showed good linearity (*R*^2^ > 0.9999) in a relatively wide concentration range. The limits of detection (LOD) and the limits of quantification (LOQ) of the six analytes were 0.20–0.91 μg/mL and 0.67–3.02 μg/mL, respectively. The results indicated that the established method was sensitive enough for the quantification of the six analytes in turmeric.

#### 2.3.2. Precision and Stability

As shown in Table 2, the established method showed good precision for the quantification of the six analytes, with the relative standard deviation (RSD) of intra- and inter-day variations less than 2%. For the stability test, the same sample solution was analyzed every 6 h for 48 h at room temperature. The results indicated that the analytes in the sample solution were stable over 48 h, with RSDs less than 2%.

#### 2.3.3. Accuracy

The recovery test was used to evaluate the accuracy of the described analytical method. The mean recovery of the six analytes ranged from 93.24–104.83%, with RSDs less than 5% (see Table 3), which indicated that the accuracy of the established method is promisingly established for quality evaluation of turmeric.

### 2.4. Quality Evaluation of Commercial Turmeric Samples

Using the calibration curve of each investigated compound, the six analytes in twelve turmeric samples were determined (Table 4). From Table 4, it was found that the levels of six individual analytes present in the samples varied considerably. The content of curcumin (at the range of 10.16 mg/g to 16.48 mg/g) in all samples was the highest among the three curcuminoids, which meets the requirement (more than 10.0 mg/g in crude material and 9.0 mg/g in processed material) of China Pharmacopoeia [1]. However, the contents of the three volatile compounds were very different among different samples. α-Turmerone (3.54 mg/g to 30.27 mg/g) was present in the highest content in the samples 3, 6, 9–12. The variety of the volatile compounds is much larger than that of the curcuminoids, which might be caused by the readily volatile property of the volatile compounds. It is well known that the quality of medicinal herbs can be influenced by many factors, such as the cultivating site, harvesting time, and post-harvest handling. Therefore, to obtain a consistent quality and efficacy of turmeric, it is suggested that all procedures involved in the production of this herb should be standardized.

## 3. Materials and Methods

### 3.1. Chemicals and Materials

HPLC grade methanol and acetonitrile were from J.T. Baker (Phillipsburg, NJ, USA). Acetic acid was purchased from Merck (Darmstadt, Germany). Ultra deionized water utilized in the study was obtained by a Milli-Q water purification system (Millipore, Billerica, MA, USA).

Turmeric samples were collected from different locations in China or purchased from local retailers in China, and were identified and authenticated as the rhizome of *C. longa* by Prof. Song-Lin Li from Jiangsu Province Academy of Chinese Medicine according to the standards of Chinese Pharmacopoeia [1]. The voucher specimens were deposited at the Institute of Chinese Medicine Science, Macau University, Macau SAR, China. The reference compound curcuminoids were isolated and purified by column chromatography on silica gel and Sephadex LH-20 columns, and three volatile compounds were isolated by the high speed counter-current chromatography (HSCCC) method from the rhizomes of *C. longa* [32]. Their structures were established by ^1^H NMR, ^13^C NMR, and MS spectral analysis. Their purities were analyzed to be all above 98% by HPLC.

### 3.2. Chromatographic Conditions

The separation was implemented on a Waters 2695 HPLC system (Waters, Milford, MA, USA), comprising a quaternary gradient pump, auto sampler, column oven, photodiode array detector, and data acquirement and processing was operated by the Waters Empower 2 software (Waters, Milford, MA, USA). The qualitative analysis was carried out on A Zorbax SB-C18 column (250 mm × 4.6 mm i.d., 5 μm) with a Zorbax SB-C18 guard column (20 mm × 4 mm, 5 μm) (Agilent Technologies, Santa Clara, CA, USA). The column temperature was set at 35 °C. The mobile phase was composed of acetonitrile (A) and water containing 0.4% (*v*/*v*) acetic acid (B). The gradient program was as follows: 45% A at 0–13 min, 45–56% A at 13–16 min, 56% A at 16–50 min, and 56–100% A at 50–55 min. The re-equilibration duration was 10 min between individual runs. The flow rate was kept at 1.0 mL/min. The injection volume was 10 μL. An online detection wavelength was selected at wavelengths of 240 nm and 430 nm.

### 3.3. Pressurized Liquid Extraction (PLE)

PLE were performed on a Dionex ASE 200 system (Dionex, Sunnyvale, CA, USA). The turmeric samples were dried at 50 °C for 24 h and then comminuted into a powder of 0.2–0.3 mm. The turmeric sample (0.5 g) was mixed with diatomaceous earth (0.5 g) and placed into an 11 mL stainless steel extraction cell. The sample was extracted under the optimized conditions: Ethanol was used as the extraction solvent, temperature was set at 100 °C; pressure was set at 1500 psi; and the sample was static extracted for 5 min by a flush volume of 60%. Then, the turmeric extracts were transferred to a 50 mL volumetric flask and diluted to volume with ethanol. All sample solution was filtered through a 0.22 μm membrane before use in the HPLC system. The above sample preparation procedure was repeated twice for each commercial turmeric sample for quantification.

### 3.4. Preparation of Standard Solution

The six reference compounds were accurately weighed and dissolved in methanol. The mixed standard solution containing all reference compounds was prepared in a 10 mL volumetric flask, diluting with methanol, and stored at 4 °C. Subsequently, the stock solution was further diluted with methanol to obtain a series of concentrations of working solutions to establish calibration curves.

### 3.5. Validation of the Quantitative Analysis

The HPLC method described in this article was validated in terms of linearity, sensitivity, precision, stability, and accuracy. Six different concentrations of working solutions were analyzed in triplicate to establish calibration curves. The calibration curves of six analytes were constructed by plotting the mean peak areas vs. the concentration of the reference compounds. The limits of detection (LOD) and quantification (LOQ) were determined by injecting a series of dilute standard solutions until a signal-to-noise ratio (S/N) of 3 and 10 was obtained, respectively.

The precision test was performed by the measurements of intra- and inter-day variability. For the intra-day precision test, turmeric samples were extracted using the PLE method and analyzed for six replicates within one day, while, for the inter-day precision test, the samples were examined in duplicates for three consecutive days. Quantities of the analytes were calculated from their corresponding calibration curves. The RSD was used to evaluate the precision.

The stability test was performed by analyzing one sample at 0, 2, 4, 6, 8, 12, 24, and 48 h, respectively. Also, the RSD was taken as the measurements of stability. The accuracy of the method was determined by a spiked recovery test at three different concentration levels. Accurate amounts of mixed standard solutions at three different concentration levels (in the range of the calibration curve) were added into 0.25 g of the sample 8 powder, and then extracted and analyzed using the method as described above. The recovery was calculated with the following equation: Recovery (%) = (amount determined − amount original)/amount spiked × 100%.

## 4. Conclusions

For the first time, a PLE and HPLC method was developed and validated to simultaneously quantify three curcuminoids (bisdemethoxycurcumin, demethoxycurcumin and curcumin) and three volatile compounds (*ar*-turmerone, *β*-turmerone, and *α*-turmerone) of the rhizome of *C. longa*. This method was successfully applied for quantification of the two types of bioactive components in twelve turmeric samples, and was clarified to be a specific, sensitive, and accurate method for the quality control of turmeric. Inconsistency of these bioactive compounds among commercial samples was observed and it is suggested that all procedures involved in the production of this herb should be standardized to ensure consistent quality and, consequently, efficacy of turmeric.

## Figures and Tables

**Table 1:** Calibration curves, limits of detection (LODs), and limits of quantification (LOQs) of the six analytes.

Analyte	Linear Equation ^a^	*R* ^2 b^	Linear Range (μg/mL)	LOD (μg/mL) ^c^	LOQ (μg/mL) ^d^
Bisdemethoxycurcumin	*y* = 101,215*x* − 75,414	0.9999	3.13–100	0.20	0.67
Demethoxycurcumin	*y* = 87,918*x* − 66,314	1.0000	1.56–100	0.26	0.88
Curcumin	*y* = 86,839*x* − 64,613	0.9999	1.56–100	0.31	1.04
*ar*-Turmerone	*y* = 26,801*x* − 33,652	1.0000	6.25–400	0.73	2.45
*β*-Turmerone	*y* = 69,449*x* − 49,841	1.0000	3.13–200	0.51	1.71
*α*-Turmerone	*y* = 35,041*x* − 46,310	1.0000	3.13–200	0.91	3.02

^a^*y*, peak area; *x*, concentration of the analytes (μg/mL); ^b^ the correlation coefficient; ^c^ limit of detection (S/N = 3); ^d^ limit of quantification (S/N = 10).

**Table 2:** Precision and stability of the six analytes.

Analyte	Precision				Stability (*n* = 8)
	Intra-Day (*n* = 6)		Inter-Day (*n* = 6)		
	Detected (μg/mL) ^a^	RSD (%) ^b^	Detected (μg/mL)	RSD (%)	RSD (%)
Bisdemethoxycurcumin	27.84 ± 0.24	0.86	27.59 ± 0.32	1.15	1.27
Demethoxycurcumin	34.07 ± 0.32	0.93	33.70 ± 0.41	1.21	1.30
Curcumin	120.94 ± 1.16	0.96	120.61 ± 0.80	0.66	0.92
*ar*-Turmerone	114.21 ± 0.75	0.66	116.30 ± 2.30	1.98	0.17
*β*-Turmerone	37.36 ± 0.23	0.62	38.22 ± 0.77	2.02	0.19
*α*-Turmerone	40.94 ± 0.45	1.10	41.37 ± 0.56	1.35	0.25

^a^ All values are mean ± S.D.; ^b^ RSD% = (S.D./mean) × 100%.

**Table 3:** Recoveries for the assay of six analytes.

Analyte	Original (mg)	Spiked (mg)	Found (mg) ^a^	Recovery (%) ^b^	RSD (%)
Bisdemethoxycurcumin	0.69	0.6	1.27	96.88	4.27
		0.7	1.34	93.24	2.43
		0.8	1.49	99.64	3.33
Demethoxycurcumin	0.84	0.6	1.44	101.14	1.79
		0.8	1.68	104.83	0.99
		1	1.88	104.12	2.13
Curcumin	3.01	2.5	5.61	104.22	3.75
		3	5.93	97.47	3.18
		3.5	6.56	101.43	1.74
*ar*-Turmerone	2.84	2.20	5.02	99.21	2.60
		2.50	5.37	101.03	2.66
		3.00	5.70	95.35	2.99
*β*-Turmerone	0.93	0.80	1.71	98.11	3.39
		0.90	1.80	97.10	3.83
		1.00	1.90	96.59	4.68
*α*-Turmerone	1.01	0.90	1.90	99.00	3.31
		1.00	2.00	99.00	3.68
		1.20	2.20	99.43	3.23

^a^ The data is presented as an average of three determinations; ^b^ Recovery (%) = (amount determined − amount original)/amount spiked × 100%.

**Table 4:** Contents of three curcuminoids and three volatile compounds in commercial samples (mg/g).

Sample	Collecting Region	BMC	DMC	C	*ar*-Turmerone	*β*-Turmerone	*α*-Turmerone
1	Songzhou, Sichuan	3.49 ± 0.06	4.98 ± 0.12	16.48 ± 0.45	12.43 ± 0.44	5.47 ± 0.21	8.19 ± 0.33
2	Songzhou-2, Sichuan	3.69 ± 0.08	3.51 ± 0.10	11.67 ± 0.23	5.33 ± 0.04	4.00 ± 0.03	9.48 ± 0.18
3	Sichuan-1	3.11 ± 0.05	4.14 ± 0.09	12.23 ± 0.54	10.51 ± 0.35	7.64 ± 0.11	18.57 ± 0.02
4	Wenshan, Yunnan	4.31 ± 0.15	4.99 ± 0.10	16.06 ± 0.17	10.38 ± 0.39	6.71 ± 0.14	15.88 ± 0.44
5	Sichuan-2	3.68 ± 0.07	4.90 ± 0.11	15.35 ± 0.51	8.01 ± 0.03	5.62 ± 0.01	12.67 ± 0.05
6	Sichuan-3	3.15 ± 0.04	2.64 ± 0.05	10.16 ± 0.13	4.74 ± 0.09	5.52 ± 0.02	16.91 ± 0.28
7	Guangzhou, Guangdong	3.79 ± 0.02	5.82 ± 0.05	10.94 ± 0.09	11.68 ± 0.18	3.50 ± 0.03	3.54 ± 0.04
8	Shaoguan, Guangdong	2.76 ± 0.06	3.35 ± 0.06	12.02 ± 0.05	11.36 ± 0.03	3.72 ± 0.02	4.04 ± 0.03
9	Zhanjiang, Guangdong	2.86 ± 0.02	3.64 ± 0.05	11.47 ± 0.38	12.93 ± 0.22	8.92 ± 0.14	21.54 ± 0.85
10	Hanzhong, Shanxi	3.63 ± 0.05	4.37 ± 0.05	14.32 ± 0.11	8.77 ± 0.05	9.77 ± 0.08	30.27 ± 0.12
11	Anguo, Hebei	5.83 ± 0.04	7.60 ± 0.09	14.91 ± 0.05	11.88 ± 0.05	8.50 ± 0.45	19.55 ± 0.34
12	Yulin, Guangxi	3.22 ± 0.03	4.50 ± 0.01	14.60 ± 0.07	11.04 ± 0.02	10.27 ± 0.10	29.16 ± 0.24

The data is presented as an average of duplicates from two individual extracts for each sample.
